# Heterogeneity in Fragile X Syndrome Highlights the Need for Precision Medicine-Based Treatments

**DOI:** 10.3389/fpsyt.2021.722378

**Published:** 2021-09-30

**Authors:** Edgard Verdura, Laura Pérez-Cano, Rubén Sabido-Vera, Emre Guney, Jean-Marc Hyvelin, Lynn Durham, Baltazar Gomez-Mancilla

**Affiliations:** ^1^Discovery and Data Science (DDS) Unit, Sociedad Limitada (STALICLA SL), Barcelona, Spain; ^2^Research Programme on Biomedical Informatics, Hospital del Mar Medical Research Institute (IMIM), Departament de Ciències Experimentals i de la Salut (DCEXS), Pompeu Fabra University (UPF), Barcelona, Spain; ^3^Drug Development Unit (DDU), Société Anonyme (STALICLA SA), Geneva, Switzerland; ^4^Department Neurology and Neurosurgery, McGill University, Montreal, QC, Canada

**Keywords:** Fragile X syndrome, precision medicine-based treatments, autism spectrum disorders, biomarkers, monogenic disease, heterogeneity

## Abstract

Fragile X syndrome (FXS) is the most frequent monogenic cause of autism or intellectual disability, and research on its pathogenetic mechanisms has provided important insights on this neurodevelopmental condition. Nevertheless, after 30 years of intense research, efforts to develop treatments have been mostly unsuccessful. The aim of this review is to compile evidence from existing research pointing to clinical, genetic, and therapeutic response heterogeneity in FXS and highlight the need of implementing precision medicine-based treatments. We comment on the high genetic and phenotypic heterogeneity present in FXS, as a contributing factor to the difficulties found during drug development. Given that several clinical trials have showed a non-negligeable fraction of positive responders to drugs targeting core FXS symptoms, we propose that success of clinical trials can be achieved by tackling the underlying heterogeneity in FXS by accurately stratifying patients into drug-responder subpopulations. These precision medicine-based approaches, which can be first applied to well-defined monogenic diseases such as FXS, can also serve to define drug responder profiles based on specific biomarkers or phenotypic features that can associate patients with different genetic backgrounds to a same candidate drug, thus repositioning a same drug for a larger number of patients with NDDs.

## Introduction: Heterogeneity Beyond Idiopathic Neurodevelopmental Disorders

Neurodevelopmental disorders (NDDs) are a group of prevalent and highly heterogeneous conditions characterized by impairment in “personal, social, academic, or occupational functioning” with onset early in development, which include autism spectrum disorder (ASD), intellectual disability (ID), attention deficit hyperactivity disorder (ADHD), communication disorders, specific learning disorders, and motor disorders (1); moreover, the definition can also include some neuropsychiatric disorders such as schizophrenia and bipolar disorder, and other neurologic disorders such as cerebral palsy or epilepsy (2). Comorbidity of two or more of these disorders is observed at rates higher than what it would be expected by chance, suggesting the existence of clusters of shared biological mechanisms (3, 4). While cooccurrence of neurological features and conditions is frequently observed, very often, NDDs also include a variety of extra-neurological clinical signs such as hypotonia, dysmorphology, cardiologic or metabolic features, as well as gastrological problems such as constipation or diarrhea, which are specially frequent in ASD and ADHD (5–7), or immunological abnormalities (8, 9). The recent advances in genotyping and sequencing technologies have propelled the identification of risk/causal genes, which has pointed to remarkable genetic heterogeneity among and within specific NDDs; for intellectual disability alone, pathogenic mutations in more than 1,000 genes have been confirmed in the SysID database (a systematic and manually curated catalog of ID-associated genes) (10). While syndromic neurodevelopmental conditions associated to mutations in one single gene are by definition genetically more homogeneous, they still involve varying degrees of phenotype and genetic heterogeneity (11). Further complexifying matters: well-defined clinical entities can be caused by mutations in several genes (as it is the case for Noonan syndrome, linked to mutations in 14 different genes) (12), while mutations occurring in a same gene can result into a wide spectrum of symptoms, as exemplified by *MECP2* mutations in Rett syndrome (13). Adjunctly, different kinds of mutations can result in different pathophysiological mechanisms, as recently exemplified by mutations in *SATB1*, in which three different kinds of variants were associated to distinct pathological consequences (14).

Fragile X syndrome (FXS) is a well-characterized NDD syndrome, caused by deficiency of FMRP (Fragile X Mental Retardation Protein, encoded by *FMR1*), an RNA-binding protein that regulates editing, translation, stability, and transport of a large number of neuronal mRNAs (15). FXS has been extensively studied in the last 30 years. It was first clinically described in 1943 as a form of X-linked inherited intellectual disability (16). In 1969, Lubs developed the chromosomal test for FXS (17), although the causal gene, *FMR1* (Fragile X Mental Retardation 1), and the mutational basis of FXS, were not discovered until 1991 (18). FXS is classically caused by an expansion of >200 CGG repeats in the promoter region of *FMR1* (hereinafter referred as full *FMR1* mutation); this leads to the promoter methylation and transcriptional silencing of *FMR1*. Nowadays, FXS represents the most frequently identified monogenic cause of ASD and inherited intellectual disability (19). But despite being a genetically well-characterized syndrome, there is considerable heterogeneity within patients with FXS, and the condition remains a high unmet medical need. This supports the necessity for deeper characterization of the population with FXS to enable the development of efficient treatments.

## Clinical and Genetic Heterogeneity in FXS

A considerably high level of clinical and genetic heterogeneity can be found in FXS. Despite being a highly recognizable syndrome from the clinical point of view, patients with FXS manifest a wide spectrum of behavioral phenotypes, although some of them such as attention/hyperactivity, hyperarousal, anxiety, and aggression episodes are commonly seen. Females with FXS show a similar spectrum of behavioral difficulties compared to males with FXS, but with milder symptoms (20). Notably, there is a strong association between FXS and autism spectrum disorder (ASD), as ~50% of FXS male and 20% female patients meet DSM-5 criteria for this disorder, and FXS is the leading monogenic cause of ASD (21). Although autistic features are not present in all individuals it is highly speculated that FXS and some groups of patients with idiopathic ASD present with shared pathophysiology, as defects in many proteins that interact with FMRP have also been associated with idiopathic ASD (15). Importantly, greater severity and lower level of functioning is associated with ASD co-morbidity in FXS (22–24). A higher prevalence of seizures, sleep problems, and co-occurring problematic behaviors, especially aggressive/disruptive behavior, is found in the pediatric population with FXS and a diagnosis of ASD than in the FXS population without ASD (19). Individuals with FXS also widely differ in level of cognitive impairment, while some males with FXS function nearly normally, and others are profoundly disabled (25). Several studies have reported “high-functioning” males with intellectual ability in the normal to borderline range (26, 27). Besides, one-third to half of females with a full *FMR1* mutation have intellectual functioning in the normal range, due to the masking effect of the normal X-chromosome *FMR1* allele (28). Numerous additional associated conditions and symptoms of variable severity can occur such as sleep disturbance, seizures, frequent otitis media, strabismus, and joint hyperlaxity (29). Interestingly, a FXS subgroup has been reported, characterized by hyperphagia, lack of satiation after meals and extreme obesity with a full, round face, small, broad hands/feet, and regional skin hyperpigmentation, referenced in literature as “Prader-Willi-like” (30, 31). This particular subgroup might point to the existence of several subgroups of patients with FXS which can be grouped based on phenotypic features and treated by targeting the corresponding underlying molecular differences. Prevalence of several conditions or symptoms associated to FXS are shown in Tables 1, 2.

**Table 1:** Reported prevalence of heterogeneous phenotypic features in FXS patients in several studies.

**Global phenotypic features**					
**Feature**	**References**	**Prevalence in FXS females**	**Prevalence in FXS males**	**Prevalence in FXS**	**Prevalence in FXS-negative controls**
Adverse response to touch on the skin	Lachiewicz et al. (32)	–	–	61.1% (22/36)	18.9% (7/37)^*^
Aortic root dilatation	Ciaccio et al. (33)	–	–	25%	–
Brisk deep tendon reflexes	Lachiewicz et al. (32)	–	–	72.7% (26/36)	37.8% (14/37)^*^
Broad forehead	Lachiewicz et al. (32)	–	–	72.2% (26/36)	67.6% (25/37)–
Curvature of the spine	Lachiewicz et al. (32)	–	–	5.6% (2/36)	2.7% (1/37)–
Curved 5th finger	Lachiewicz et al. (32)	–	–	63.9% (23/36)	48.6% (18/37)–
Difficulty touching tongue to lips	Lachiewicz et al. (32)	–	–	75.9% (22/29)	27.6% (8/29)^*^
Difficulty pronouncing “puh–tuh–kuh”	Lachiewicz et al. (32)	–	–	72.4% (21/29)	46.2% (12/26)–
Difficulty moving the extended tongue from side to side	Lachiewicz et al. (32)	–	–	53.6% (15/28)	17.2% (5/29)^*^
Difficulty pronouncing “linoleum”	Lachiewicz et al. (32)	–	–	86.2% (25/29)	73.1% (19/26)–
EEG anomalies	Ciaccio et al. (33)	–	–	74%	–
Elongated/narrow face	Lachiewicz et al. (32)	–	–	83.3% (30/36)	45.9% (17/37)^*^
	Lubala et al. (34)			72.18% (109/151)	19.53% (533/2,728)^*^
	Ciaccio et al. (33)	–	–	83%	–
Epilepsy / Seizures	Lachiewicz et al. (32)	–	–	8.6% (3/35)	13.5% (5/37)–
	Berry–Kravis et al. (35)	4.8% (1/23)	13.3% (15/113)	11.7% (16/136)	–
	Berry–Kravis et al. (36)	6% (19/304)	14% (154/1,090)	12,41% (173/1,394)	–
	Kidd et al. (37)	3.2% (*n* = 62)	12.1% (*n* = 198)	10% (*n* = 260)	–
	Bailey et al. (38)	2.7% (7/259)	1.84% (18/976)	2.02% (25/1,235)	–
	Ciaccio et al. (33)	–	–	10 – 20%	–
	Symons et al. (39)	94% (*n* = 51)	81.8% (*n* = 436)	–	–
Flat feet	Lachiewicz et al. (32)	–	–	69.4% (25/36)	62.2% (23/37)–
	Yuskaitis et al. (40)	–	–	50% (75/150)	–
	Lubala et al. (34)	–	–	70.27% (26/37)	37.39% (43/115)^*^
	Ciaccio et al. (33)	–	–	29 – 69%	–
Gastrointestinal problems	Kidd et al. (37)	7% (*n* = 62)	12% (*n* = 198)	10.8% (*n* = 260)	–
	Ciaccio et al. (33)	–	–	31%	–
Hallucal crease	Lachiewicz et al. (32)	–	–	82.9% (29/35)	29.7% (11/37)^*^
Hyperextensible joints	Lachiewicz et al. (32)	–	–	100% (36/36)	75.7% (28/37)^*^
	Lubala et al. (34)	–	–	68.18% (150/220)	25.44% (849/3,336)^*^
Hand calluses	Lachiewicz et al. (32)	–	–	27.8% (10/36)	2.7% (2/37)^*^
Highly arched palate	Lachiewicz et al. (32)	–	–	94.4% (34/36)	70.3% (26/37)–
Horizontal palmar creases or distal axial triradii	Lachiewicz et al. (32)	–	–	25% (9/36)	13.5% (5/37)–
History of eye problems	Lachiewicz et al. (32)	–	–	45.7% (16/35)	21.6% (8/37)–
History of cleft lip/palate	Lachiewicz et al. (32)	–	–	2.8% (1/36)	0% (0/37)–
Hypotonia	Lachiewicz et al. (32)	–	–	72.2% (26/36)	48.6% (18/37)–
History of allergies	Lachiewicz et al. (32)	–	–	37.1% (13/35)	32.4% (12/37)–
History of spine curvature	Lachiewicz et al. (32)	–	–	2.8% (1/36)	2.7% (1/37)–
History of hernias	Lachiewicz et al. (32)	–	–	8.3% (3/36)	5.4% (2/37)–
History of > five ear infections/recurrent otitis media	Lachiewicz et al. (32)	–	–	97.2% (35/36)	91.9% (34/37)–
	Kidd et al. (37)	45.8% (*n* = 62)	54.7% (*n* = 198)	52.6% (*n* = 260)	–
	Ciaccio et al. (33)	–	–	47 – 97%	–
Inability to close eyes on request	Lachiewicz et al. (32)	–	–	14.5% (4/27)	0% (0/29)–
Joint hypermobility/Excessive laxity of the joints	Ciaccio et al. (33)	–	–	50%	–
	Yuskaitis et al. (40)	–	–	57% (85/150)	–
Large and prominent ears	Lubala et al. (34)	–	–	83.9% (173/206)	21.86% (756/3,458)^*^
	Ciaccio et al. (33)	–	–	72 – 78%	–
	Lachiewicz et al. (32)	–	–	72.2% (26/36)	35.1% (13/27)^*^
Large testicles/Macroorchidism	Lachiewicz et al. (32)	–	–	62.9% (22/35)	29.7% (11/37)^*^
	Lubala et al. (34)	–	–	69.61% (129/181)	9.98% (291/2,915)^*^
	Ciaccio et al. (33)	–	–	63 – 95%	–
	Ciaccio et al. (33)	–	–	94%	–
Low birth weight	Kidd et al. (37)	12.7% (*n* = 62)	7.2% (*n* = 198)	8.6% (*n* = 260)	–
Macrocephaly/Head circumference > 50th centile	Lachiewicz et al. (32)	–	–	80.6% (29/36)	62.2% (23/37)–
	Ciaccio et al. (33)	–	–	81%	–
Mitral click	Lachiewicz et al. (32)	–	–	2.8% (1/36)	0% (0/37)–
Mitral valve anomalies	Ciaccio et al. (33)	–	–	3 – 12%	–
Mitral Valve prolapse	Kidd et al. (37)	1.7% (*n* = 62)	0.5% (*n* = 198)	0.8% (*n* = 260)	–
Motor tics	Kidd et al. (37)	6.7% (*n* = 62)	5.4% (*n* = 198)	5.7% (*n* = 260)	–
Nystagmus	Ciaccio et al. (33)	–	–	5 – 13%	–
Obesity	Ciaccio et al. (33)	–	–	53 – 61%	–
Obstructive sleep apnea	Kidd et al. (37)	7.1% (*n* = 62)	7.2% (*n* = 198)	7.2% (*n* = 260)	–
Ocular abnormalities	Lachiewicz et al. (32)	–	–	27.8% (10/36)	21.6% (8/37)–
Pale blue eyes	Lubala et al. (34)	–	–	57.14% (28/49)	7.25% (23/317)^*^
Prominent helices	Lachiewicz et al. (32)	–	–	66.7% (24/36)	40.5% (15/37)–
Prominent jaw	Ciaccio et al. (33)	–	–	80%	–
Pectus excavatum	Lachiewicz et al. (32)	–	–	50% (18/36)	29.7% (11/37)–
Pectus excavatum	Ciaccio et al. (33)	–	–	50%	–
Plantar crease	Lubala et al. (34)	–	–	85.71% (84/98)	22.91% (162/707)^*^
Refractive errors	Ciaccio et al. (33)	–	–	17 – 59%	–
Simply formed helices	Lachiewicz et al. (32)	–	–	27.8% (10/36)	13.5% (5/37)–
Strabismus	Kidd et al. (37)	12.9% (*n* = 62)	17.5% (*n* = 198)	16.4% (*n* = 260)	–
	Ciaccio et al. (33)	–	–	8 – 40%	–
Sleep problems	Kidd et al. (37)	29.8% (*n* = 62)	26% (*n* = 198)	26.9% (*n* = 260)	–
Spine deformity	Ciaccio et al. (33)	–	–	6 – 9%	–
Scoliosis	Yuskaitis et al. (40)	–	–	6.6% (10/150)	–
Skin soft and velvety	Lubala et al. (34)	–	–	88.37% (38/43)	5.24% (95/1,811)^*^
Soft skin over dorsum of hand	Lachiewicz et al. (32)	–	–	100% (35/35)	73% (27/37)^*^
Transverse palmar crease/Sydney lines	Lubala et al. (34)	–	–	26% (30/115)	9.77% (104/1,064)^*^

* *Studies in which a significative difference was found between FXS patients and FXS-negative controls*.

**Table 2:** Reported prevalence of heterogeneous neurobehavioral features in FXS patients in several studies.

**Neurobehavioral features**					
**Feature**	**Study**	**Prevalence in FXS females**	**Prevalence in FXS males**	**Prevalence in FXS**	**Prevalence in FXS-negative controls**
Autism/Autistic-like behavior	Symons et al. (41)	6.17% (16/259)	4.71% (46/976)	5.02% (62/1,235)	–
	Symons et al. (39)	34% (*n* = 51)	55.4% (*n* = 436)	–	–
	Lubala et al. (34)	–	–	76.05% (162/213)	24.7% (854/3,457)^*^
	Budimirovic et al. (42)	1/5 (20%)	12/26 (46%)	–	–
	Kaufmann et al. (21)	18% (*n* = 237)	51% (*n* = 237)	42% (*n* = 237)	–
	Lewis et al. (43)	–	–	10/44 (22%)	–
Attention problems	Ciaccio et al. (33)	–	–	74 – 84%	–
	Symons et al. (41)	25.86% (67/259)	8.6% (84/976)	12.22% (151/1,235)	–
	Lubala et al. (34)	–	–	79.13% (91/115)	48.07% (511/1,063)^*^
	Symons et al. (39)	81.6% (*n* = 51)	87.4% (*n* = 436)	–	–
ADHD	Lubala et al. (34)	–	–	75.3% (122/162)	55.20% (870/1,576)^*^
Anxiety Disorder	Ciaccio et al. (33)	–	–	58 – 86%	–
	Budimirovic et al. (42)	–	–	100% (31/31)	–
Depression	Ciaccio et al. (33)	–	–	8 – 12%	–
	Symons et al. (41)	8.49% (22/259)	1.22% (12/976)	2.75% (34/1,235)	–
	Symons et al. (39)	29.2% (*n* = 51)	13.5% (*n* = 436)		
Developmental delay	Symons et al. (41)	24.71% (64/259)	9.83% (96/976)	12.95% (160/1,235)	–
Family history of intellectual disability	Lachiewicz et al. (32)	–	–	69.4% (25/36)	32.4% (12/37)^*^
	Lubala et al. (34)	–	–	80.97% (166/205)	23.61% (807/3,418)^*^
Gaze avoidance/ poor eye contact	Lachiewicz et al. (32)	–	–	83.3% (30/36)	51.4% (19/37)^*^
	Lubala et al. (34)			86.33% (139/161)	34.32% (517/1,506)^*^
Hand flapping	Lubala et al. (34)	–	–	58,59% (75/128)	29% (404/1,391)^*^
Hand–biting	Lubala et al. (34)	–	–	39.13% (45/115)	20.52% (218/1,062)^*^
Hyperactivity	Symons et al. (41)	11.58% (30/259)	6.76% (66/976)	7.77% (96/1,235)	–
	Ciaccio et al. (33)	–	–	50 – 66%	–
	Lubala et al. (34)	–	–	74.07% (120/162)	52.6% (829/1,576)^*^
	Symons et al. (39)	38.8% (*n* = 51)	71.9% (*n* = 436)		
	Symons et al. (41)	21.62% (56/259)	7.17% (70/976)	10.2% (126/1,235)	–
Perseverative speech	Lubala et al. (34)	–	–	66.45% (107/161)	46.04% (675/1,466)^*^
Previous diagnosis of intellectual disability	Lachiewicz et al. (32)	–	–	91.4% (32/35)	64.9% (24/37)^*^
Self-injurious behavior	Symons et al. (41)	3.86% (10/259)	4.2% (41/976)	4.12% (51/1,235)	–
Sleep problems	Ciaccio et al. (33)	–	–	30%	–
Tactilely defensive	Lubala et al. (34)	–	–	65% (108/166)	19.12% (626/3,274)^*^

* *Studies in which a significative difference was found between FXS patients and FXS-negative controls*.

This important degree of phenotypic heterogeneity in FXS probably mirrors a heterogeneous genetic background and the cellular-level involvement of various signaling pathways co-regulated by FMRP, such as PI3K and mTOR pathways (44). Moreover, genetic background plays an important role as shown in animal models (which would result in different patterns of expression of other proteins, including FMRP-interacting proteins) (45, 46), as well as (a) genetic consequences of variation on FMRP function at different levels including FMRP expression, and (b) FMRP effect on other genes mRNA transcripts by regulation of splicing, translation (through ribosome stalling), and RNA stability through the recognition of mRNA codon bias and N6-methyladenosine (m6A) modifications (15). In addition, individuals with FXS might show mosaicism at two different levels: (1) CGG repeat lengths, with some cells harboring fully expanded mutation alleles and other cells harboring more benign alleles; and (2) methylation levels, with some cells containing methylated *FMR1* alleles and other cells containing unmethylated *FMR1* alleles. It is estimated that nearly half of individuals carrying the full *FMR1* mutation exhibit some sort of size and/or methylation mosaicism (47) (Table 3). Novel methods have improved detection of these alleles, which previously could only be detected by Southern blot (50). Size mosaicism, which is thought to arise due to CGG repeat instability, is normally observed as a combination of full mutation (>200 repeats) alleles with premutation alleles (>55) or rarely, even normal alleles. Methylation mosaicism can be observed in the form of unmethylated alleles, either showing a full mutation or a premutation allele. Both types of mosaicism will support the production of some FMRP, so individuals with size and /or methylation mosaicism might have less severe cognitive and behavioral defects than a patient with a full mutation and a completely methylated *FMR1* promoter, and in whom FMRP is markedly reduced or absent (19). Several authors have reported that male patients having full mutation with complete methylation had the lowest IQ scores and greatest physical involvement, in comparison to mosaic cases, although other studies have not observed this correlation (48, 51, 52). Correlation of degree of size and/or methylation mosaicism with other phenotypic features (seizures, hyperactivity, and autism) has been more difficult to establish. In a recent paper evaluating a cohort of male and female patients with FXS, male children carrying full *FMR1* mutation and expressing some degree of *FMR1* mRNA due to incomplete methylation had significantly higher Autism Diagnostic Observational Schedule (ADOS) severity scores, compared to individuals with FXS carrying full *FMR1* mutation but with completely silenced *FMR1*. However, this association should be replicated in additional cohorts (53). In female premutation carriers, a study found that *FMR1* mRNA levels increase as the number of CGG repeats increases, suggesting that due to skewed X inactivation, mRNA levels tend to normalize in females when the number of CGG repeats increases, making clinical-genetic correlations more difficult to establish (54). While larger studies are needed, the expression levels of FMRP and methylation status of the *FMR1* gene have been correlated with cognitive ability (positive correlation for FMRP levels, negative correlation for methylation) (55), whereas little correlation between CGG repeat number and cognition is thought to exist. Given that residual levels of FMRP expression explain in part the heterogeneity in the FXS phenotype, the integration of diagnostic genomic data with *FMR1* mRNA measuring assays and more accurate FMRP profiles could clarify the relationships between genotypes, mRNA/protein expression and patient phenotypes. Deciphering these links would both improve disease prognostics and be useful to stratify patients with FXS for clinical trials (56).

**Table 3:** Mosaicism features in FXS patient cohorts.

**Study**	**Features**
Nolin et al. (45)	**148 patients (males)** Full mutation/full methylation **→** 87/148 (59%) Mosaic pattern (full mutation/premutation) **→** 61/148 (41%)
Merenstein et al. (48)	**218 patients (males)** Full mutation/full methylation **→** 160/218 ~73% Full mutation partial methylation **→** 12/218 ~6% males Mosaic pattern (full mutation/premutation) v 46/218 ~ 21% males
Rousseau et al. (49)	**1,051 patients (485 males, 283 females)** Full mutation/full methylation **→** 425 males (87.6%), 268 females (94.6%) Mosaic pattern (full mutation/premutation) **→** 60 males (12.3%), 15 females (5.3%)

In a very recent study, which assessed quantification of methylation, DNA, RNA, and FMRP protein levels in buccal and blood samples, methylation mosaicism was estimated to be slightly over 50%. Molecular-neurobehavioral correlations confirmed the inverse relationship between overall severity of the FXS phenotype and decrease in FMRP levels. Co-occurrence of FXS with an autism diagnosis correlated significantly with 2-fold lower levels of FMRP specially in younger age- and IQ-adjusted males, compared to FXS without ASD, and patients with severe Intellectual Disability had even lower FMRP levels (42). In the same study, while Budimirovic et al. (42) also showed a high level of agreement in regard to FMRP protein levels between blood and buccal samples, but these findings have not always been replicated, and discordant presence of mutations between different tissues such as blood and skin have been reported (57–59). In parallel, it has been shown that repeat number can vary in different tissue types from the same individual carrying premutation alleles, suggesting that numbers obtained from blood mononuclear cells may not always directly translate to the brain. These results can complicate efforts to use blood results for clinical trial inclusion/exclusion criteria (60), as they suggest that FMRP levels in patients' cortical brain tissue might differ from levels observed in blood, at least for some FXS patients. Evidences of correlation between blood and brain FMRP levels in the individual subjects are limited; in particular, a study comparing brain and blood suggested that males with full mutations also have a certain level of mosaicism in brain tissue (61, 62). Further studies using blood and post-mortem brain samples will be required to shed light on this matter.

## Diversity of Responses To Therapeutic Treatments

Despite two decades of preclinical work and several clinical trials targeting at least 10 different mechanisms associated with FXS pathogenesis (19), candidate drugs have been unsuccessful in reaching primary endpoints, and there are still no approved treatments to address the core symptoms of FXS. Well-studied pathophysiological mechanisms of FXS include excessive glutamatergic signaling, endocannabinoid system impaired signaling, voltage-gated ion channel dysfunction, GABAergic system inhibition, or excess of protein translation activity. While preclinical studies of drugs targeting these mechanisms in FXS mouse models (such as the widely used FMR1-KO model) provided positive results, translation to therapeutic use in human patients have not achieved the expected efficacy outcomes. Nevertheless, several clinical trials are ongoing, and several past clinical trials conducted in FXS did show clinical benefit in subsets of patients, providing hope for future drug development studies (63).

FMRP plays a critical role in mGluR (metabotropic glutamate receptor)-dependent long-term depression and its absence causes dysregulated synaptic function due to excessive and persistent protein synthesis in postsynaptic dendrites (64). Accordingly, in two clinical trials with mGlu5 receptor antagonists (fenobam, mavoglurant), which target an excessive activity of mGluR signaling downstream due to loss of inhibitory control by FMRP, improvement was observed in six and seven subjects out of 12 and 30 patients, respectively. In the fenobam study, a calmed behavior with improvement in eye contact, ability to interact, anxiety and/or motor overactivity was observed in nine out of 12 patients, pointing to an even higher rate, although further studies were not performed afterwards (65). In the initial mavoglurant clinical trial, seven out of 30 responder patients identified in a *post-hoc* analysis, had improved Aberrant Behavior Checklist—Community Edition scores (ABC-C) associated to complete *FMR1* promoter methylation and no detectable *FMR1* messenger RNA, while no improved response was shown in 18 patients with partial promoter methylation (66). These encouraging results prompted the design of two large IIb clinical trials (double—blind, placebo—controlled, performed in parallel, *n* = 175, 139), in which patients were also stratified by methylation status. Nevertheless, these studies did not report data on efficacy scores, nor evaluated the presence of positive responder patients (67). After negative results in the Phase III study, Novartis announced the discontinuation of the mavoglurant FXS development program (68). However, despite lack of success in past clinical trials, further studies continue to support a possible role of *FMR1* promoter methylation to stratify patients (69, 70).

On the other hand, in 2012 a first phase II clinical trial with arbaclofen (a GABA_B_ receptor agonist) was performed based on successful preclinical model studies (71). Although no differences from placebo on the primary endpoint (irritability scores) were found, secondary outcome measures were associated with significant improvement. Using a novel ABC—Social Avoidance (ABC-SA) scale validated for the assessment of FXS, arbaclofen treatment was also associated with an overall beneficial effect. Furthermore, a *post-hoc* analysis focused on a subgroup of 27 subjects with more severe social impairment showed improvements in several scales compared to placebo treatment. The results were also more robust among subjects who met Diagnostic and Statistical Manual of Mental Disorders-Fourth Edition (DSM-IV) and Autism Diagnostic Interview-Revised (ADI-R) criteria for autistic disorder. Significantly more subjects were responders on the CGI-I scale when receiving arbaclofen vs. placebo (35 vs. 18% overall; 50 vs. 6% autism) in the autism subgroup, although again the ABC-C Irritability was not sensitive to these effects. Arbaclofen was then tested in two parallel phase 3 studies (randomized, double—blind, placebo—controlled, *n* = 125 and 172) in adults and children (35). These two studies did not show benefit for arbaclofen over placebo for any measure (including the primary objective, showing efficacy reducing the ABC-CFX Social Avoidance score). Nevertheless, the child study showed that the highest dose group was associated to a benefit over placebo on ABC-CFX Irritability subscale and Parenting Stress Index, and results showed a trend toward improvement in social avoidance and hyperactivity subscales and CGI-I. Although additional studies with a larger cohort on higher doses would be required to confirm this finding, these results suggested potential dose- and age-related effects as well as a possible optimization of primary study endpoints, pointing toward a potential benefit of arbaclofen in future optimized clinical trials. Double-blind, randomized, placebo-controlled studies in FXS are also highly recommended to properly assess the sensitivity and specificity of clinical endpoints (63, 72, 73).

Clinical trials have also been performed for other drugs with limited success. Although not reaching primary outcomes, secondary endpoints or *post-hoc* analyses pointed to a considerable positive responder fraction of patients in several of them (Table 4), which suggest a potential benefit of these drugs that should be targeted and improved in future clinical trials. Only very recently, a promising phase-2 crossover study using phosphodiesterase-4D inhibitors (to increase cAMP production levels, which are reduced in patients with FXS) on a cohort of 30 FXS male adult patients showed improvement on cognition and daily function, while also meeting the primary objectives of safety and tolerability (78). Although these results shall be validated in future larger clinical trials, this study accounts for the importance of addressing cohort variability through the selection of meaningful endpoints capturing inter-individual variability.

**Table 4:** FXS clinical trials showing positive responder results.

**References**	**Drug**	**Phase**	**Positive responders %**	**Primary endpoint**	**Scales used to define or measure improvement in responders**	**Negative responders**
Berry-Kravis et al. (65)	Fenobam (mGluR5 antagonist)	IIa, open-label study	50% (6/12: 4/6 males and 2/6 females)	-	PPI (improved over test–retest controls)	No adverse effects reported
Jacquemont et al. (66)	Mavoglurant (mGluR5 antagonist)	IIa	23.33% (7/30 males)	ABC-C (not attained)	ABC-C of selected patients after *post-hoc* analysis	No neurobehavioral adverse effects reported (24/ 30 mild to moderate fatigue and headache)
Berry-Kravis et al. (71)	Arbaclofen (GABA_B_ receptor agonist)	II	47.6% (10/21 patients with increased social impairment, most of them males) vs. 8.7% (placebo)	ABC-I (not attained)	Defined by CGI-I, ABC-_LSW_	No neuro behavioral adverse effects reported (8% cases of sedation and of headache)
Berry-Kravis et al. (35)	Arbaclofen (GABA_B_ receptor agonist)	III	35% in children patients, vs. 21% (placebo)	ABC-_CFX_ (not attained)	Defined by CGI-I, ABC-_CFX_	Irritability, agitation, anxiety, hyperactivity (45 vs. 40% controls in adults, ~36 vs. 34% in children), other extra neurological features
Erickson et al. (74)	Acamprosate	III open-label study	75% (9/12 subjects)	CGI-I	CGI-I	irritability, repetitive behavior
Berry-Kravis et al. (75)	Lithium	IIa	86% (13/15, ABC-C), 86% (13/15, CGI), 80% (12/15, VAS),	ABC-C Irritability (not attained)	ABC-C, CGI, VAS	Irritability, appetite changes, bed wetting, constipation or diarrhea, headache, polydipsia, polyuria, sleep problems, tiredness, vomiting, high TSH
Paribello et al. (76)	Minocycline	IIa (open-label)	63% (12/19)	ABC-C	ABC-C, CGI, VAS	Minor diarrhea, seroconversion to a positive ANA
Greiss Hess et al. (77)	Sertraline		52% (13/25 sertraline) vs. 44% (12/27 control) improved symptoms	CGI-I, MSEL-EL	MSEL-EL, *post-hoc* analysis	Upper respiratory infection, diarrhea, and gastrointestinal issues
Berry-Kravis et al. (78)	BPN14770	II, two-way crossover study	NA	Safety, tolerability	National Institutes of Health-Toolbox Cognition Battery (NIH-TCB) and Test of Attentional Performance for Children (KiTAP)	There was no TEAE (treatment-emergent adverse events) judged by the investigator to be at least possibly related to treatment with BPN14770.

*ABC-C, Aberrant Behavior Checklist–Community Edition; ABC-I, ABC–Irritability; ABC-LSW, ABC—Lethargy/Social Withdrawal; ABC-CFX, ABC- Fragile X specific; MSEL-EL, Mullen Scales of Early Learning, Expressive Language; ET, eye tracking; CGI-I, Clinical Global Impression-Improvement; PPI, Prepulse Inhibition; VAS, Visual Analog Scale*.

## Discussion: The Need and Potential Implications of Precision Medicine in FXS and Related Syndromes

Although past efforts in clinical trials with FXS have mainly resulted in lack of success at meeting primary endpoints, these works have not been unfruitful. These large studies have built a base for future studies in FXS and other NDDs, in which several improvements should be incorporated, such as: use of optimized primary outcome measures (both neurobehavioral and related to cognition/language), discovery of novel prognosis and progression markers, administration of drugs from an earlier age and possibly longer times, performance of well-powered studies (as clinical trials with a lower number of participants were invariably positive in *post-hoc* analyses), and a better analysis and translation of preclinical mouse model studies to clinical studies in humans (63). A particular point of relevance for the future design of successful clinical trials in FXS is the characterization and further identification of subgroups of patients that respond to a specific drug treatment. While some studies have focused on establishing lists of minimal features to be screened to diagnose patients with FXS (32, 34), less focus has been put on the identification of subgroups of patients with FXS according to their phenotypic or molecular characteristics. Improved patient stratification would most likely help to pair pharmacologic agents with patients most likely to respond positively to such therapeutic treatments.

Besides *FMR1* methylation levels and selection on isolated clinical features (such as high-functioning individuals or Prader-Willi-like subphenotype mentioned above), other stratification strategies based on precision medicine have been suggested and might be implemented in the future. A recent work reported the use of structural brain growth as a marker to identify clinically significative subgroups. Using topological data analysis on T1-weighted anatomical MRI data from 42 FXS children patients, researchers identified two previously unknown large subgroups of patients. *Post-hoc* analyses between these groups demonstrated that one group was consistently higher functioning on cognition, adaptive functioning, and autism severity scores. As pointed by the authors, anatomical MRI data analysis might become a useful method to define subtypes within other neuropsychiatric disorders (79). In another recent study, electronic health records from more than one million people were mined to investigate health characteristics of individuals clinically diagnosed with FXS. This resulted into (1) the identification of previously unnoticed significative co-occurring health conditions in patients with FXS (heart and circulatory disorders, medication side effects, and among others), and (2) the development of a predictive model to identify patients with FXS in the general population without using any genetic data, successfully identifying cases 5 years prior to clinical diagnosis of FXS (80). While this AI-assisted diagnosis method was instrumental to identify cases in the general population prior to the onset of more severe symptoms (80), no computational methods have been oriented toward stratifying patients with FXS into more homogenous subgroups, which is the first step needed to enrich future clinical trials in FXS with responder patients. Recently, the use of patient-derived induced pluripotent stem cells was proposed to model the disease in a patient-specific manner and to develop new therapeutic opportunities (81); nevertheless, to our knowledge, there is still no clinical trials in FXS involving therapeutic treatments developed using patient-derived induced pluripotent stem cells.

Accurate stratification of patients is expected to be crucial for the development of efficient drug treatments in FXS. Early applications of systems biology driven *in silico* drug repositioning in FXS were conducted without specific focus on genetic heterogeneity (HealX drove the advancement of a FXS repurposing discovery effort in collaboration with FRAXA) (82). In order to overcome this limitation, systems biology and precision-medicine based computational aided modeling are emerging in the NDD space both in academic and industry setting. These approaches offer new potential for novel subgroup characterization and further identification of FXS and other NDD patients with stronger biological potential to respond to specific drug candidates. For instance, STALICLA's DEPI platform was recently used to identify subsets of clinical features that significantly correlate with the molecular responses induced by arbaclofen in cellular models, which could support the identification of patients predicted to improve under arbaclofen's treatment. An observational study involving patients with FXS which had participated in previous clinical trials with arbaclofen is currently ongoing, with the main goal to provide clinical validation for this defined subgroup of patients with FXS. Importantly, other NDD patients (without a FXS diagnose) might qualify to fit into this target population and potentially benefit from the same compound.

In summary, well-defined NDD syndromes such as FXS (the most frequent monogenic cause of intellectual disability and ASD among NDDs) constitute a first step to switch from a behavioral to a molecular-centered based diagnosis. Precision medicine in FXS will continue to be necessary to (1) define more precisely subgroups of patients for clinical trials, and (2) define drug responder profiles that can associate patients with different genetic backgrounds to a same candidate drug. Importantly, these targeted populations of patients might expand beyond the FXS indication.

## Author Contributions

All authors listed have made a substantial, direct and intellectual contribution to the work, and approved it for publication.

## Conflict of Interest

All authors work at Stalicla.

## Publisher's Note

All claims expressed in this article are solely those of the authors and do not necessarily represent those of their affiliated organizations, or those of the publisher, the editors and the reviewers. Any product that may be evaluated in this article, or claim that may be made by its manufacturer, is not guaranteed or endorsed by the publisher.
